# Research on Mechanical and Carbonization Properties of Hybrid Fiber Iron Tailings Concrete Based on Deep Learning

**DOI:** 10.1155/2022/3475679

**Published:** 2022-06-09

**Authors:** Wenbo Zheng, Sheliang Wang, Yang Qu, Kangning Liu, Liangwei Jia

**Affiliations:** ^1^School of Civil Engineering, Xijing University, Xi'an 710123, China; ^2^School of Civil Engineering, Xi'an University of Architecture and Technology, Xi'an 710055, China

## Abstract

Iron tailings sand is a kind of mineral waste, and open-air storage is a common treatment method for iron tailings, which not only has a huge impact on the ecological environment but also occupies a lot of land resources. Therefore, the preparation of high-ductility fiber reinforced iron tailings concrete and its application in practical engineering structures have good application prospects. This paper is based on the deep learning research on the mechanical and carbonization properties of hybrid fiber iron tailings concrete. Therefore, tailings sands with different substitution rates, single-mixed steel fiber, and mixed steel-PVA fiber concrete were prepared in this paper. Its compressive strength, split tensile strength, axial compressive strength, elastic modulus, strain, and carbonization depth were tested. Through the existing concrete compressive stress-strain curve equations, the nonlinear calculation of each group of concrete compressive stress-strain curve equations in this paper is carried out, some parameters are determined, and the carbonation depth equation is established. The results show that, with the increase of tailings content, the properties of concrete increase first and then decrease and the addition of fibers can effectively improve the strength, elastic modulus, peak strain, and carbonization depth of concrete. However, with the increase of PVA fiber content, its performance enhancement effect decreased.

## 1. Related Introduction

In China, the comprehensive utilization of iron tailings sand began in the 1990s. After years of experimental research, a relatively mature conclusion has been achieved in the use of iron tailings sand for the preparation of iron tailings concrete. The preparation, mechanical properties, and durability of tailings concrete were further researched and developed. In the process of iron ore mining, a large amount of solid waste will be produced. Processing tailings waste into building materials is the most effective way to absorb mineral waste and has high ecological benefits and engineering value. Due to the high compressive strength, rough texture, and angular shape of iron tailings particles, iron tailings-made concrete has better ITZ properties and durability than ordinary concrete. By using mechanical activation to improve the pozzolanic activity and hydration performance of iron tailings, secondary hydration reaction occurs with cement to improve concrete strength. A large number of experimental studies have shown that, with the increase of iron tailings replacement rate, the workability of concrete decreases, which may be due to the high water absorption and specific surface area of iron tailings material, which leads to a decrease in workability. Although iron tailings as fine aggregates can improve the compressive strength of concrete, considering that it will reduce the workability of concrete, it is generally recommended to use iron tailings not exceeding 30% as fine aggregates. Lv conducted a comparative study by completely replacing ordinary aggregates with iron tailings in dam concrete with different grades.

It can be seen from the above literature that scholars have little research on the carbonization properties of iron tailings and hybrid fibers. Therefore, this paper studies its mechanical properties such as compressive strength, axial compressive strength, elastic modulus, stress-strain, and carbonization depth and provides a basis for the carbonization performance of iron tailings hybrid fibers in a wider range of applications [1–10].

## 2. Related Tests

### 2.1. Materials

#### 2.1.1. Cementitious Material

The cementitious material used in this test consists of two parts, Qinling brand ordinary Portland cement and fly ash with a strength grade of P·O 42.5 R. The minerals and physical properties of the two materials are shown in Tables 1 and 2, respectively.

#### 2.1.2. Aggregate

The aggregate is composed of natural crushed stone and fine aggregate, and the fine aggregate is river sand and natural sand. The physical properties of the tested aggregate are shown in Table 3.

#### 2.1.3. Fiber

The fibers used in the test are steel fibers and PVA fibers, and the performance indicators of the two fibers are shown in Table 4.

### 2.2. Mixing Ratio

This test is divided into ordinary concrete (NC), iron tailings concrete (TAC), steel fiber concrete (TAC-S), and steel-polyvinyl alcohol fiber concrete (TAC-S-P) according to the iron tailings replacement rate and fiber content. . The replacement rate of iron tailings is 30%, 50%, and 100%. In most studies, the optimal dosage of steel fiber is roughly 1.5%. The effects of polyvinyl alcohol fiber content of 0, 0.3%, 0.6%, and 0.9% on the mechanical properties and carbonation durability of concrete were discussed [11].

This article refers to the requirements of the “Ordinary Concrete Mix Proportion Design Regulations” (JGJ55-2011) and other specifications. After the previous trial and adjustment, the concrete mix design is shown in Table 5. Among them, the water-cement ratio of each group of mixing ratios is 0.4, and the sand ratio is 0.37.

^*∗*^1 is for number naming; take “T30-S1.5-P0.3 as an example;” T stands for iron tailings, S stands for steel fiber, P stands for PVA fiber, the number “30” means 30% iron tailings replacement rate, and the number “0.3″ means that the PVA fiber content is 0.3%.

### 2.3. Preparation of Test Pieces

According to the mixing ratio of 5 in Table 5, 12 samples were prepared in each group, including 9 cube samples of 100 mm × 100 mm × 100 mm; the compressive strength of the cubes at carbonization ages of 0 d, 7 d, 14 d, 28 d, and 56 d was tested, respectively, and three prisms with a sample size of 100 mm × 100 mm × 300 mm were tested for axial compressive strength, stress-strain curve, elastic modulus, and stress-strain [12].

### 2.4. Test Instruments and Programs

The carbonization experiment was carried out in a carbonization test chamber, the CO2 concentration was kept at (20 ± 3)%, the humidity was controlled at (70 ± 5)%, the temperature in the chamber was controlled at (20 ± 2)°C, and the carbonization ages were 7, 14, 28, and 56 days.

## 3. Analysis of Test Results Based on Deep Learning

### 3.1. Cube Compressive Strength

#### 3.1.1. Compressive Strength of Iron Tailings Concrete before and after Carbonization

**Figure 1:**
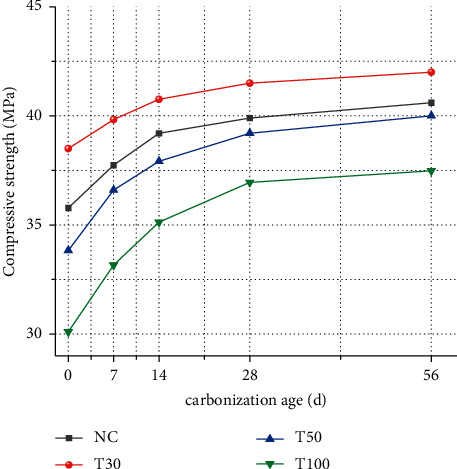
Compressive strength of iron tailings concrete.

Figure 1 shows the effects of iron tailings replacement rate and carbonation age on compressive strength under different compressive strength. It can be seen from the test results that when the replacement rate of iron tailings is increased, the concrete strength increases first and then decreases. When the replacement rate of iron tailings is 30%, the compressive strength of concrete reaches the highest, the strength increases by 7.6%, the replacement rate is 50% and 100%, and the strength decreases by 5.8% and 18.9%, respectively.

With the increasing carbonation age, the compressive strength of concrete always maintains a stable growth trend. When the carbonation age reaches 7d, the increase of compressive strength is the largest, and the compressive strength increases by 5.5%, 3.5%, 8.2%, and 10.2%, respectively. With the increase of age, the growth of carbonation compressive strength gradually stabilized.

When the concrete carbonizes, the chemical composition and pore structure inside the matrix change. CO2 reacts with Ca (OH)_2_ to form water-insoluble calcium carbonate. Calcium carbonate fills the cemented pores and microcracks inside the concrete, weakens the subsequent diffusion of CO_2,_ and increases the density and strength of the concrete [13, 14].

#### 3.1.2. Compressive Strength of Fiber Iron Tailings Concrete before and after Carbonization

**Figure 2:**
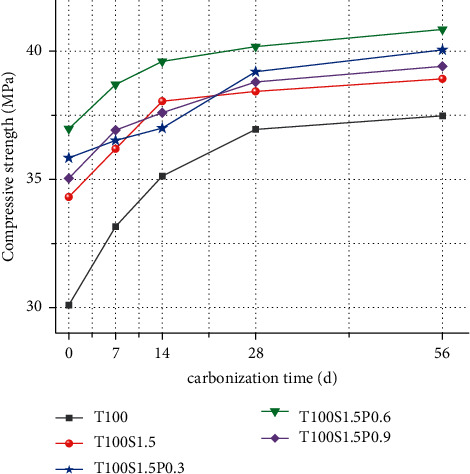
Compressive strength of fiber reinforced concrete.

Figure 2 studies the effect of fiber content and carbonization age on compressive strength, and fiber addition has a positive effect on concrete strength. With the increase of PVA fiber content, the strength of concrete increases at first and then decreases, and the strength of concrete mixed with fiber is greater than that of steel fiber alone. Fiber reinforced concrete strength increased by 14%, 19%, 22.8%, and 16.4%, respectively.

Carbonation has little effect on the strength of fiber reinforced concrete, because the addition of fibers improves the internal structure of the concrete, making it difficult for CO2 to penetrate into the concrete. After carbonization for 7d, the compressive strength of concrete increased by 5.5%, 1.9%, 4.7%, and 5.4%, respectively.

The improvement of fiber reinforced concrete strength is mainly due to two aspects of crack resistance and toughening. The anticracking effect of fibers depends on the distribution of fibers in concrete. When only steel fibers are mixed into concrete, since the density of steel fibers is greater than that of the matrix, it will sink under the action of self-weight and vibration, resulting in steel fibers in the concrete. The uneven distribution in the concrete can easily cause local stress concentration during compression, resulting in premature failure of the specimen. The increase in toughness is due to the fiber bridging-debonding-slip failure mode, which generates more dissipated energy. There are hydroxyl groups in the molecular chain of PVA fiber, which can form a strong bond between the fiber and the concrete matrix. A large number of fine PVA fibers will increase the surface area in contact with the concrete matrix, which can better transmit stress through cracks. After adding PVA fiber, a complex three-dimensional fiber network is formed with the random distribution of steel fiber. The steel fiber can inhibit the large cracks in the concrete, and the PVA fiber inhibits the early plastic shrinkage and drying shrinkage microcracks. And as a steel fiber hybrid carrier, the steel fiber is evenly dispersed in the matrix, which produces a good synergy with the steel fiber, produces a positive hybrid effect, and improves the strength of the concrete.

With the increase of PVA fiber content, it is relatively difficult to disperse too much PVA fiber, and there will be obvious agglomeration phenomenon, resulting in the formation of new weak areas where there are no fibers or less fibers in the concrete. The intervention of fibers causes the redistribution and transfer of concrete stress. If the distribution of this stress cannot achieve a true equilibrium, new stress concentrations may be formed, resulting in cracking in the area where fibers are lacking [15].

### 3.2. Cube Splitting Tensile Strength

#### 3.2.1. Splitting Tensile Strength of Iron Tailings Concrete before and after Carbonization

**Figure 3:**
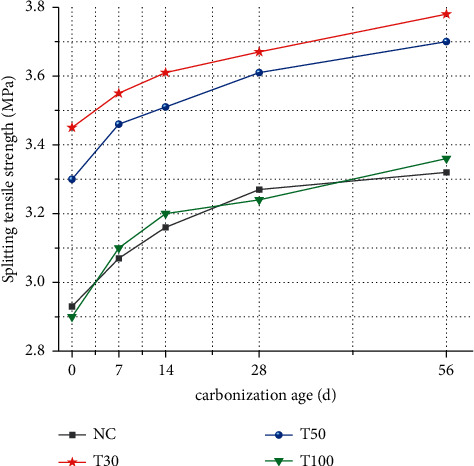
Splitting tensile strength of iron tailings concrete.

Figure 3 investigates the effect of different iron tailings substitution rate and carbonization age on splitting tensile strength. It can be seen from the figure that, with the increase of iron tailings substitution rate, the splitting tensile strength of concrete increases first and then decreases. The tensile strength is the highest when the substitution rate is 30%, and the splitting tensile strength of T30, T50, and T100 is the highest. The strength increased by 17.7%, 12.6%, and -1%, respectively. With the increase of carbonization age, the overall splitting tensile strength is in a steady growth trend, and the tensile strength of carbonized 28d increases by 11.6%, 6.4%, 9.4%, and 11.7%, respectively.

Tensile strength is an important index to determine the crack resistance in concrete design, and it is also a key parameter to indirectly determine the bond strength of each component of concrete. In this experiment, 30% of iron tailings was the optimal replacement level, the concrete had the densest filling structure, and the bonding performance between the aggregate and the cement mortar was improved. As the content of iron tailings increases, the bonding properties between aggregates deteriorate, resulting in a decrease in tensile strength.

Carbonization makes the splitting tensile strength value of concrete more complicated. With the increase of carbonization age, the carbonization strength value fluctuates greatly. The addition of iron tailings has a great influence on the strength value of the low carbonization period. When the carbonization cycle is long, the effect is relatively small. For example, when the dosage is 100%, the strength value increases by 6.9%, 3.2%, 1.25%, and 3.7%, respectively, at the carbonization age of 7d, 14d, 28d, and 56d. The reason is that as the carbonization time increases, the hydration products gradually fill the pores in the concrete after carbonization, so that there are fewer channels for CO2 to enter and leave the concrete, which reduces the impact of carbonization [16–20].

#### 3.2.2. Splitting Tensile Strength of Fiber Reinforced Concrete before and after Carbonization

**Figure 4:**
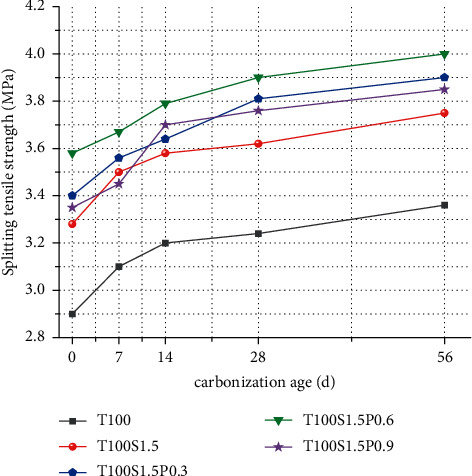
Splitting tensile strength of fiber reinforced concrete.

It can be seen from Figure 4 that the addition of fibers has a significant effect on improving the splitting tensile strength value of concrete and the concrete strength is increased by 13.1%, 17.2%, 23.4%, and 15.5%, respectively.

The effect of single-doped steel fiber on the splitting tensile properties of iron tailings concrete can be explained as follows: due to the rough surface of iron tailings, the bonding performance between steel fibers and aggregates is enhanced, and when the concrete is loaded, the steel fibers are difficult to be pulled out or break, thereby increasing the tensile strength value. Second, steel fibers can inhibit the development of cracks. Third, the tensile force is transmitted to the steel fibers through the concrete matrix, and the bridging effect between the steel fibers and the concrete makes up for the defects of the concrete matrix. During the splitting and tensile failure of concrete, steel fibers absorb a lot of energy.

Mixing fibers can significantly improve the tensile strength value of concrete, and the mixing effect is better than that of steel fibers alone. When steel fibers are mixed with PVA fibers, PVA fibers form a fiber grid, evenly dispersed in concrete. It has a good synergistic effect with steel fiber, and a positive hybrid effect occurs, which greatly improves the tensile strength value of concrete. With the increase of PVA fiber volume ratio, the tensile strength value of hybrid fiber reinforced concrete showed an increasing trend. This is because, with the increase of PVA fiber content, the fiber spacing is relatively low in concrete, which can effectively limit the development of primary cracks caused by factors such as segregation and shrinkage, thereby improving the tensile strength value. When the fiber content is more than 0.6%, the tensile strength value of concrete will be reduced. According to the analysis of the compressive test above, if too much PVA fiber is added, there will be obvious agglomeration, so that the fibers are not uniformly distributed in the concrete, and the fiber distribution is too sparse or too dense at the splitting interface, resulting in a larger concentrated stress reducing the tensile strength value.

With the increase of carbonization age, the value of splitting tensile strength has been increasing. It may be because in the early stage of carbonization CO2 in the air penetrates into the internal pores of the concrete and dissolves the water in the pores. The carbonization reaction of the hydration product generates calcium carbonate, which fills part of the pores, and the bridging effect of the PVA fiber and the matrix makes the expansion stress distribution of the concrete pores more uniform. In the later stage of carbonization, the generated calcium carbonate fills the internal pores of the concrete, and the addition of fibers also reduces the cracks of the concrete, and it is difficult for CO2 to enter the concrete again, so the effect of increasing the tensile strength value in the later stage of carbonization is not obvious.

When the steel fiber is used alone, due to the high density and thick diameter of the steel fiber, the volume ratio of the steel fiber contained in the same volume of concrete is larger, the fiber spacing of the steel fiber is significantly higher than that of other fibers, and CO2 easily enters the concrete. After adding PVA fiber, steel fiber and PVA fiber have a good synergistic effect, reducing the fiber spacing between fibers, and its effect on capillary thinning and blocking is more obvious, thereby reducing the pore channel of CO2 in the concrete, so the mixing effect is due to the single mixing of steel fibers [21, 22].

### 3.3. The Mechanism of Fiber Crack Inhibition

The strength, deformation, and failure of concrete are related to the propagation of cracks. The essence of concrete failure is the expansion and penetration of original microcracks until macroscopic cracks are produced, which eventually lead to concrete instability and failure. Fiber can effectively prevent the generation and development of cracks and improve the crack resistance of concrete. The main reason is that when fibers are added to the concrete, they are evenly distributed in the concrete. As a result of fiber and concrete, there is a very strong grasp force; when the crack is further expanded, it can be timely blocked by adjacent fiber, in order to prevent crack expansion and improve the resistance of concrete cracking. The fiber forms a uniform three-dimensional chaotic support system in concrete, which can weaken the plastic shrinkage of mortar and disperse the shrinkage energy to the fiber filaments, thus effectively enhancing the toughness of concrete and reducing the cracks caused by the shrinkage of mortar. At the same time, the formation of uniform chaotic support system can effectively prevent aggregate segregation and ensure the uniform seepage of mortar in the early stage, so as to prevent the formation of settlement cracks. When the concrete bears the larger compression load and crack, the crack tip will produce stress concentration, and the crack will continue to expand under the continuous action of load. When the crack tip intersects with the fiber, the fiber can offset part of the stress and effectively prevent the crack from developing into a penetrating crack. With the decrease of fiber spacing, the stress concentration at the crack tip is gradually relieved or even disappears.

### 3.4. Uniaxial Compression

#### 3.4.1. Stress-Strain Curve

**Figure 5:**
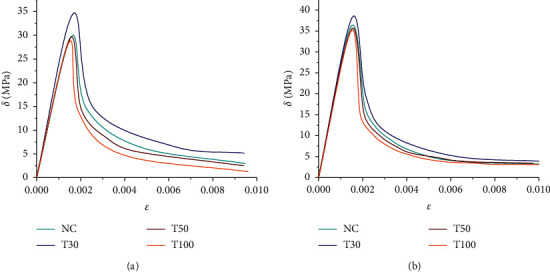
Effect of carbonization on the stress-strain curve of iron-added tailings concrete. (a) Carbide 0d. (b) Carbide 56d.

**Figure 6:**
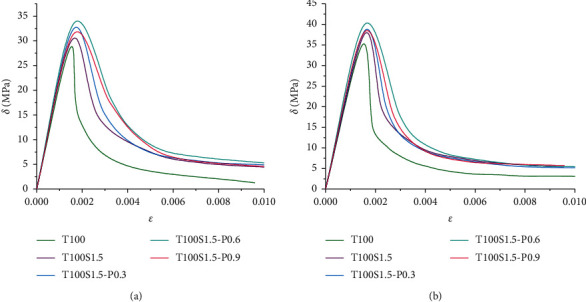
Effect of carbonization on the stress-strain curve of fiber reinforced concrete. (a) Carbide 0d. (b) Carbide 56d.

The stress-strain relationship is a key parameter to measure the mechanical properties of concrete, and it is the main basis for analyzing the bearing capacity and deformation of concrete structures. The stress-strain curve of concrete includes an ascending segment and a descending segment, which is a comprehensive macroscopic response of the basic characteristics such as the appearance and development of plastic strain, the generation of microcracks, internal defects and damage, ultimate strength, residual strength, and deformation. In this paper, the stress-strain curves of concrete in carbonation 0d (28d natural curing) and 56d are studied by adding different performance reinforcing materials, as shown in Figures 5 and 6, respectively.

From Figure 5, the stress-strain curves of the tailings concrete before and after carbonization are essentially the same, with a small change in the ascending section and a large change in the descending section, indicating that the amount of tailings and carbonation has a greater impact on the descending section of the concrete. The descending section of concrete mixed with a small amount of iron tailings is almost the same as that of ordinary concrete. The larger the amount of tailings added, the steeper the descending section. With the increase of the amount of tailings, the stiffness of concrete decreases, the ability of resisting elastic deformation weakens, and the brittleness increases. The longer the carbonization period, the steeper the descending section of the curve, indicating that the plastic deformation capacity is reduced and the failure process is more rapid.

It can be seen from Figure 6 that the addition of fibers increases the peak stress, peak strain, and residual stress of the stress-strain curve. Compared with ordinary concrete, the behavior after cracking changes, and the curve after cracking is relatively flat, indicating that the brittleness of the material is reduced and the area of the lower region increases, indicating that more energy can be absorbed. Compared with the single-doped steel fiber, the stress drop rate of the hybrid fiber after the peak is slower, the residual stress is higher, and the toughness and deformation properties of the specimen are significantly improved, which proves that the PVA fiber has a positive effect on improving the ductility of concrete. With the increase of PVA fiber content, the peak stress decreased slightly, and the slope of the downward branch of the stress-strain curve decreased slightly with the increase of PVA fiber content. Similar to the effect of adding iron tailings, the longer the carbonization period, the steeper the descending section, and the more increased the concrete brittleness.

#### 3.4.2. Axial Compressive Strength

**Figure 7:**
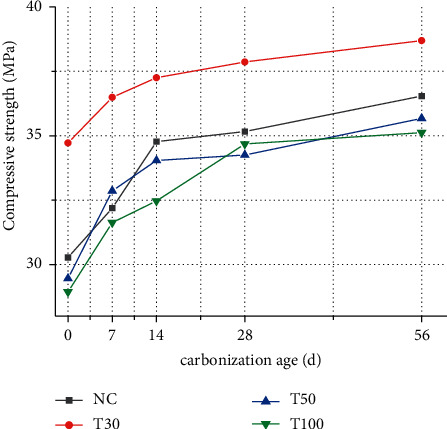
Axial compressive strength of iron tailings concrete.

**Figure 8:**
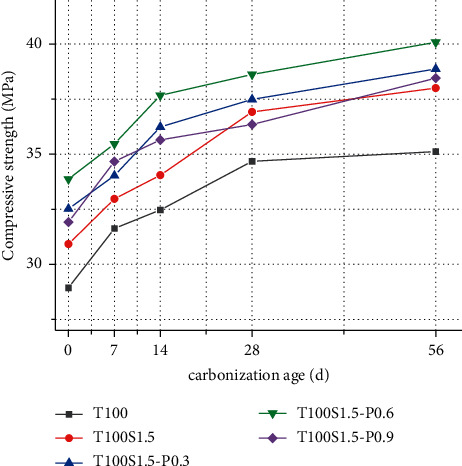
Axial compressive strength of fiber reinforced concrete.

The axial compressive strength of each mix ratio is shown in Figures 7 and 8. Similar to the cube compressive strength discussed previously, the addition of iron tailings and fibers effectively increases the axial compressive strength of concrete, and, with carbonization and the increase of age, the axial compressive strength increases steadily. The strength of tailings concrete increased by 14.7%, -3.3%, and -4.4%, and the strength of fiber concrete increased by 6.8%, 12.4%, 17%, and 10.3%, respectively. It can be seen from the above that the compressive strength of concrete decreases with the increase of PVA fiber content, which is the same as the compressive strength of cubes.

#### 3.4.3. Modulus of Elasticity

**Figure 9:**
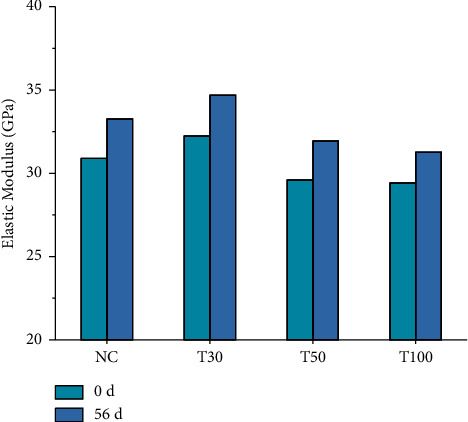
Elastic modulus of iron tailings concrete.

*(1) Elastic Modulus of Iron Tailings Concrete before and after Carbonization*.  Figure 9 shows the elastic modulus of iron tailings concrete at different ages before and after carbonization. It can be seen that the elastic modulus of iron tailings increases first and then decreases. The compressive strength of concrete is achieved by homogenizing the constituent materials and their strength. Therefore, the elastic modulus of concrete increases with the increase of concrete strength. The elastic modulus represents the deformation resistance of the material under stress, that is, the stiffness of the material. The addition of tailings reduces the stiffness of the concrete, and the ability of resisting elastic deformation is weakened, resulting in varying degrees of reduction in elastic modulus. With the extension of carbonation age, the elastic modulus of concrete with each mix ratio increases, possibly due to the formation of water-insoluble CaCO3 in the carbonization process to fill the pores of the concrete and improve the compactness of the concrete, thereby increasing the elastic modulus of the concrete.

**Figure 10:**
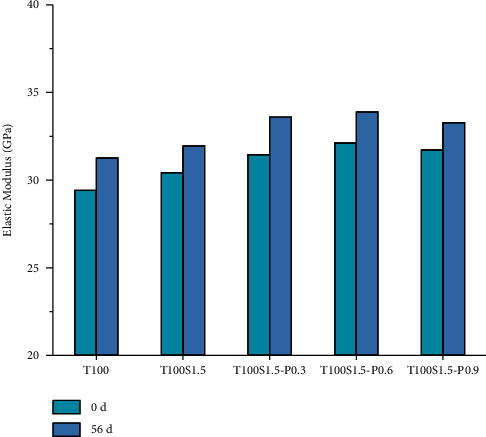
Modulus of elasticity of fiber reinforced concrete.

*(2) Elastic Modulus of Fiber Reinforced Concrete before and after Carbonization*.  Figure 10 shows the elastic modulus of fiber concrete at different ages before and after carbonization. It can be seen that the addition of fibers increases the elastic modulus of concrete. This is because the addition of fibers delays the formation of microcracks, while the elastic modulus decreases with the increase of PVA fiber content, which may be caused by the uneven distribution of excessive fibers in the concrete. When the PVA fiber content was 0.6%, the elastic modulus increased the most, which increased by 11.8%, 10%, and 9.2%, respectively. When the content was 0.9%, the elastic modulus increased by 8.2%, 9.3%, and 7.8%, respectively. Like tailings concrete, with the increase of carbonization age, the elastic modulus of fiber reinforced concrete increases, among which tailings concrete increases the most, followed by single-mixed steel fiber and hybrid fiber. Fiber reinforced concrete has less effect.

#### 3.4.4. Peak Strain

**Figure 11:**
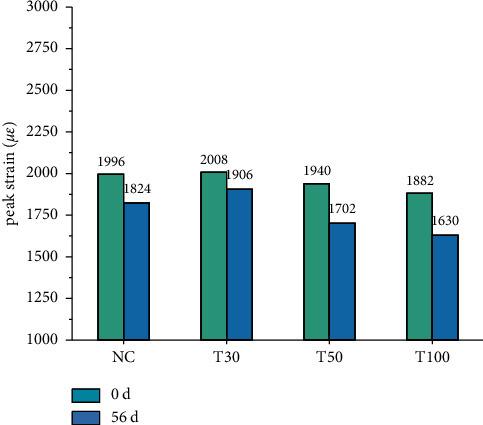
Peak strain of tailings concrete.

*(1) Peak Strain of Iron Tailings Concrete before and after Carbonization*.  Figure 11 shows the peak strain of tailings concrete. It can be seen that the peak strain of ordinary concrete is small, because the mechanical behavior of ordinary concrete is brittle in nature, and the postpeak performance decreases during the compression process due to the limited lateral strain capacity of the material. With the increase of tailings content, the peak strain first increased and then decreased. The addition of low-volume tailings optimizes the internal pore structure of the concrete and slightly increases the peak strain of the concrete. The increase of the tailings content is not conducive to the compact formation of the concrete, which increases the brittleness of the concrete and causes the concrete to break more rapidly, resulting in a decrease in the peak strain. . Although the compressive strength of concrete increases after carbonization, the brittleness of concrete increases and the peak strain decreases.

**Figure 12:**
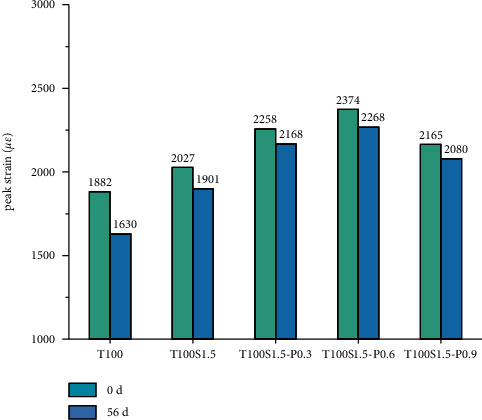
Peak strain of fiber concrete.

*(2) Peak Strain before and after Carbonization of Fiber Reinforced Concrete*. It can be seen from Figure 12 that the addition of fibers greatly increases the peak strain of concrete and the peak strain reflects the deformation ability of the specimen under the ultimate strength. The main function of fibers is to improve the postcompression performance of concrete, and fibers will continue to prevent the expansion of cracks after the concrete is compressed, so that the concrete can continue to withstand higher strains. In addition, the fibers constrain the formation of axial cracks in concrete and also increase the strain of peak stress significantly. Compared with ordinary concrete, the descending section of the stress-strain curve can act as a bridge and continue to bear the load due to the existence of fibers, so the descending section is more gentle. The peak strains increased by 7.7%, 20%, 26%, and 15%, respectively, because the addition of high-ductility PVA fibers increased the deformation of concrete before the ultimate compressive load and had a positive confounding effect with steel fibers. When the PVA fiber content is 0.9, the peak strain decreases, which may be due to the poor dispersion of more PVA fibers in the matrix.

## 4. Related Discussion

Based on the test results of deep learning, relevant discussions are mainly made from the carbonation depth of iron tailings concrete and the carbonation depth of fiber reinforced concrete. The main point of view is that, with the increase of carbonization age, the carbonation depth of concrete increases. Moreover, the carbonation depth of concrete also showed a state of first increase and then decrease, but with the increase of PVA fiber content the carbonation depth of concrete increased, showing a decreasing and then increasing trend.

### 4.1. Carbonation Depth of Iron Tailings Concrete

**Figure 13:**
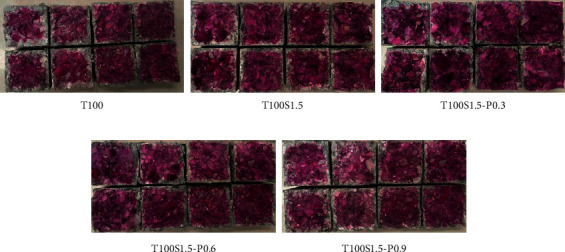
Concrete carbonation depth.

As shown in Figure 13, with the increase of carbonization age, the carbonation depth of concrete increases. With the increase of iron tailings replacement rate, the concrete carbonation depth first decreases and then increases. When the iron tailings content is 30%, the carbonation depth is the smallest.

(1) $$\begin{array}{c} \text{Ca}{\left(\text{OH}\right)}_{2}{+\text{CO}}_{2}⟶{\text{CaCO}}_{3}{+\text{H}}_{2}\text{O} \end{array}$$

(2) $$\begin{array}{c} \left(3\text{CaO}\cdot {2\text{SiO}}_{2}\cdot {3\text{H}}_{2}\text{O}\right){+3\text{CO}}_{2}⟶\left({3\text{CaCO}}_{3}\cdot {2\text{SiO}}_{2}\cdot {3\text{H}}_{2}\text{O}\right) \end{array}$$

Iron tailings are low-activity mineral admixtures with small particle size, which can exert the microaggregate effect, fill the pores between the matrix particles, make the concrete more compact, and be beneficial to the carbonization resistance of the concrete. And iron tailings have potential hydraulic and pozzolanic activity. With the increase of carbonization age, the activity of iron tailings will undergo secondary hydration reaction with Ca(OH)_2_, the main hydration product of cement, to generate hydrated silicic acid. Calcium and calcium aluminate hydrate improve the internal pore structure of concrete, thereby improving the compactness of concrete. Another reason may be that iron tailings have higher water absorption than river sand and adding an appropriate proportion of iron tailings reduces the relative humidity inside the concrete, which is not conducive to the entry of CO2. With the increase of the replacement rate of iron tailings, the secondary hydration reaction of iron tailings leads to a large consumption of Ca(OH)_2_, the hydration product of cement, which reduces the alkalinity of the hydration solution and causes the carbonization and neutralization of the concrete to be shortened, thus making the concrete carbonization and neutralization shorter. The anticarbonation performance decreased.

### 4.2. Carbonation Depth of Fiber Reinforced Concrete

As shown in Table 6, the addition of fiber significantly improves the carbonization resistance of concrete. The same as the previous law, the carbonization resistance of hybrid fiber is better than that of single-doped steel fiber. With the increase of PVA fiber content, the carbonization depth of concrete shows an increasing trend after decreasing.

The anticarbonation performance of fiber reinforced concrete is mainly reflected in two aspects: filling internal pores and reducing cracks. After a certain amount of steel fibers are mixed into the concrete, a complex three-dimensional chaotic support system is formed inside the matrix, which inhibits the subsidence of the aggregate and makes the mortar distribution more uniform, thereby improving the compactness of the concrete; at the same time, the steel is distributed in the concrete. The fibers make the capillary pores in the mortar smaller, and the capillaries are thinned or even blocked. In addition, when the concrete shrinks, the steel fiber can bear more shrinkage stress, which enhances the bonding force between the aggregate and the mortar and inhibits the formation of microcracks and the expansion of macrocracks. However, as CO2 continues to diffuse, it may lead to corrosion of steel fibers, and the formation of rust will destroy the bond between fibers and mortar, thus providing conditions for the penetration and diffusion of CO2.

Mixing steel-PVA fibers in concrete will further improve the carbonization resistance of concrete. Due to the high density and thick diameter of steel fibers, the spacing of steel fibers in concrete is too large, and the grid effect formed by steel fibers in concrete is weakened. After adding PVA fibers, the fiber spacing becomes smaller, and the fiber network formed by fiber bridging can better hinder the expansion of pores and cracks in the concrete. On the other hand, PVA fiber is a relatively hydrophilic fiber, and a large amount of water will accumulate on the surface of the fiber, which creates conditions for cement hydration, inhibits early plastic shrinkage and drying shrinkage cracks, reduces its cracking temperature, and improves concrete crack resistance.

With the increase of PVA fiber content, a large number of disordered distribution of PVA fibers in concrete will hinder the cement mortar from filling the pores of coarse aggregate, resulting in the increase of pores in the concrete. When the amount of PVA fiber increases, the mortar required to wrap the fiber is relatively reduced, which will cause concrete agglomeration during the mixing process and reduce the density of the concrete. Microscopically, there are weak interfaces intertwined between fibers and concrete. With the increase of fiber content, the number of weak interfaces in concrete increases, which provides conditions for the penetration of CO_2_.

## 5. Conclusion

Based on in-depth learning, this paper studies the mechanical and carbonation properties of concrete with different iron tailings replacement rates, single steel fiber, and mixed steel-PVA fiber concrete. Through the analysis of the test structure, the following conclusions are drawn:

1. After adding iron tailings, its compressive strength, splitting tensile strength, axial compressive strength, elastic modulus, and peak strain are enhanced in varying degrees, and the carbonation depth is reduced. With the increase of substitution rate, the performance enhancement effect shows a downward trend, but after the addition of fiber the failure form of concrete presents ductile failure, and its performance has been improved in all aspects.
2. Through the existing concrete compressive stress-strain curve equation, the nonlinear calculation of each group of concrete compressive stress-strain curve in this paper is carried out, and some parameters are determined. The calculated results are in good agreement with the experimental results.
3. With the increase of carbonation age, its compressive strength, splitting tensile strength, axial compressive strength, elastic modulus, and carbonation depth increase, the peak strain decreases, and the brittleness of concrete increases.

## Figures and Tables

**Table 1:** Chemical composition of cement and fly ash.

Quality score	SiO_2_	Al_2_O_3_	CaO	MgO	Fe_2_O_3_	TiO_2_	SO_3_	Other
Cement	55.7	42.5	0.4	—	0.3	0.9	—	0.2
Fly ash	52.97	29.96	3.66	1.52	7.98	—	0.65	3.26

**Table 2:** Physical properties of fly ash.

Physical properties of fly ash (%)				
Moisture	Loss on ignition	Water demand ratio	Fineness (45 umsquare hole sieve allowance)	Density (g/cm^3^)
0.29	3.52	93	12	2.11

**Table 3:** Physical properties of fine aggregates.

Aggregate type	Apparent area (kg/m^3^)	Loose packing(kg/m^3^)	Void ratio (%)	Waterabsorption (%)	Mud content (%)	Crushindicator (%)	
Thin bone material	River sand	2689	1655	—	0.5	3.1	—
	Iron tailings sand	2745	1824	8.7	2.9	19.53	—

**Table 4:** Physical properties of fibers.

Fiber type	Length (mm)	Diameter (um)	Aspect ratio	Density (g/cm^3^)	Tensile strength (MPa)	Elastic modulus (GPa)
Steel fiber (SF)	16	220	72.73	7.8	2500	210
Polyethylene fiber (PVA)	12	24	500	0.97–0.98	3000	160

**Table 5:** Design of concrete mix ratio.

Serialnumber	Blockcoding	Water-cementratio	Cementitious material		Coarse aggregate		Fine aggregate	Fibertype	Water	Waterreducer
			Cement	Fly ash	Stone	Riversand	Iron tailingssand	SF	PV	
1	NC	0.4	440	110	1103	656	0	0	0	220
2	T30	0.4	440	110	1103	459.2	196.8	0	0	220
3	T50	0.4	440	110	1103	328	328	0	0	220
4	T100	0.4	440	110	1103	0	656	0	0	220
5	T100-S1.5	0.4	440	110	1103	0	656	117	0	220
6	T100-S1.5-P0.3	0.4	440	110	1103	0	656	117	2.94	220
7	T100-S1.5-P0.6	0.4	440	110	1103	0	656	117	5.88	220
8	T100-S1.5-P0.9	0.4	440	110	1103	0	656	117	8.82	220

**Table 6:** Carbonation depth of fiber reinforced concrete.

Test number carbonization age	0	7	14	28	56
T100-S1.5	0	5.3	7.34	8.5	9.7
T100-S1.5-P0.3	0	4.32	6.27	7.58	8.1
T100-S1.5-P0.6	0	3.98	5.52	6.95	7.24
T100-S1.5-P0.9	0	4.77	6.8	8.11	8.36

## Data Availability

The dataset can be accessed upon request.
